# Life cycle assessment of polyphenols extraction processes from waste biomass

**DOI:** 10.1038/s41598-020-70587-w

**Published:** 2020-08-12

**Authors:** George Barjoveanu, Oana-Alexandra Pătrăuțanu, Carmen Teodosiu, Irina Volf

**Affiliations:** Department of Environmental Engineering and Management, “Cristofor Simionescu” Faculty of Chemical Engineering and Environmental Protection, “Gheorghe Asachi” Technical University of Iasi, 73, D. Mangeron Bd, 700050 Iasi, Romania

**Keywords:** Environmental sciences, Environmental chemistry, Environmental impact

## Abstract

Waste biomass from forestry and wood processing industries is a source to obtain fine chemicals, and its processing is a good example of circular economy, but it generates secondary environmental impacts. The main objective of this study was to analyse the environmental performances of laboratory scale processes for polyphenols extraction from spruce bark by means of life cycle assessment (LCA) and to simulate and evaluate the scale-up possibilities of the most favourable alternative. The assessed extraction processes were: a classic Soxhlet extraction using ethanol as solvent (SE), a high-temperature extraction in 1% NaOH solution (NaOH-SLE) and an ultrasound assisted extraction process (UAE). The functional unit was 1 mg of extracted polyphenols, measured as gallic acid equivalents (mg GAE)/g spruce bark. The life cycle inventory has included specific laboratory scale operations and extraction processes (infrastructure and transport processes were not considered). Life cycle impact assessment was performed with ReCipe 2016 at midpoint. For all extraction processes, the environmental profiles were dominated by the electricity use for heating and this has generated the highest impacts in most of the impact categories, followed by the production and use of ethanol as solvent. For the ultrasound assisted extraction, a scale-up scenario has proven that by raising capacity to a 30 L extraction vessel and by changing the heating source to a biomass-fired boiler, environmental impacts may be greatly diminished. The paper discusses also the uncertainty of lab-scale generated data for LCA. A sensitivity analysis has proven that for this case, the energy efficiency of different lab-scale equipment induce acceptable degrees of uncertainty for the LCA results.

## Introduction

Biomass has been considered among the most important renewables, with the greatest economic growth potential in the future. Biomass represents the only natural resource that can be converted into both high value-added products and energy. Therefore, the demand for biomass is increasing worldwide. Consequently, there is a growing need to better understand how biomass can be valorised sustainably, what are the biomass flows in the economy and how the increased pressure on natural resources can be adjusted with environmental and economic sustainability in Europe and globally^1^. Particularly, by-products from forestry and wood processing industry appear to be the most important and promising feedstock for recycling and for closing product life cycles, in accordance with the circular economy principles. Currently, the main route for wood bark valorization is the direct energy production by incineration at the woodmill, given its relatively high energy content (high heating value of 20.4–25.1 MJ/kg for resinous bark), but due to reasons like heterogeneity and high ash content this option is not so economically feasible^2^. The other main valorisation routes refer to different bio-refinery processes developed for the production of chemicals. The high lignin and extractives contents make wood bark a poorer source for sugars production, compared to wood, but it is considered a very promising source of chemicals and materials like: polyphenols^3^ including tannins^2^ and antioxidants^4^, foams and syngas^5^.

In this context, the spruce bark (*Picea Abies*) can be a source of fine chemicals, especially polyphenols, oligo- and polysaccharides which are separated as extractible using conventional and non-conventional extraction techniques.

The conventional extraction methods (maceration, percolation, reflux extraction and Soxhlet extraction) commonly applied to solid matrices have disadvantages when compared to greener techniques. The extraction method must be simple, fast, economic, with large applicability and with low environmental impacts. Therefore, in recent years, newer and sophisticated techniques such as microwave‐assisted extraction (MAE), accelerated solvent extraction (ASE), supercritical fluid extraction (SFE) and ultrasound assisted extraction (UAE) have been developed. These processes which are considered more environmentally friendly (“green”) due to the reduced operational time, reduced use of chemicals, better yield and better extract quality have been critically reviewed in the last decade^6–10^.

Polyphenols extraction from wood bark, as any waste or by-product valorisation technology, induces secondary environmental impacts and associated costs due to chemicals and energy consumption. To evaluate the feasibility of such processes it is necessary to perform a critical sustainability (or at least environmental) assessment to compare the reduction in primary impacts (environmental benefices) caused by valorisation with its secondary impacts and tertiary impacts (generated by the new products).

Life cycle assessment is a well-established and standardized method used to identify and quantify environmental impacts of products and processes along their life cycles, by using an input–output approach^11,12^. Conventionally LCA has been successfully used to evaluate existent large-scale processes and product life cycles. Relatively recently, new research approaches have been tested in the field of fine chemicals and green synthesis to consider waste treatment^13^, emerging technologies^14^, novel materials^15^ and innovative processes and scale-up^16^, multi-criteria comparison of complex systems^17^, to name just few methodological challenges related to biorefinery processes.

In this field, LCA has been mainly used to evaluate full-scale bioenergy production (mainly bio-ethanol^18^, biogas^19,20^, jet fuel^21^) and to a lesser extent the production of chemicals like polysaccharides^5^ or tannins^22^.

In the field of small-scale solvent extraction processes, LCA was primarily used to investigate the environmental impacts of bio-fuel production processes, especially by using algae systems^23–25^, and for bio-active compounds production, like: pharmaceuticals^26^, carotenoids ^27^.

There are only few LCA studies that have approached the environmental impacts of polyphenols extraction from bark. For example, Ding et al.^22^ have evaluated the environmental impacts of different scenarios related to tannin extraction from spruce bark (hot and cold water extraction) and have concluded that increasing the tannin yield (by increasing the extraction steps) leads to increased environmental impacts. This analysis was focused on energy-related impacts which were strongly linked to the structure of the electricity mix.

In another study, Vauchel et al.^28^ have compared by LCA the efficiency of different polyphenols extraction methods from chicory grounds and have found out that impacts are mainly related to extraction temperature increase and the use of solvents.

Most studies on environmental performance assessment of wood waste biorefinery processes focus on full-scale applications given the high quantities in which these by-products are produced. However, smaller-scale operations for the production of fine chemicals are also important from an environmental point of view, given the wider range of chemicals used, and the energy quantities used in these processing operations.

The main objective of this study is to analyse the environmental performances of some extraction processes designed to separate polyphenols from spruce bark applied at laboratory scale. These include two conventional processes: a classic Soxhlet extraction using ethanol as solvent (SE), a high-temperature solid liquid extraction in 1% NaOH solution (NaOH-SLE) and one non-conventional (“green”) process: ultrasound assisted extraction (UAE). The UAE was performed at 50 °C (UAE-50) and at 25 °C (UAE-25). Also, for comparison purposes, the UAE process was studied for different particle sizes of bark (UAE-PS) and a blank extraction (NO-UAE) was done as well. The assessment was completed by means of LCA in order to highlight both their benefits and environmental burdens. This study points out the secondary environmental impacts associated to these extraction methods considering all process parameters, material and energy inputs and outputs. Furthermore, various scenarios have been investigated in order to decrease the environmental impacts and to enhance the scale-up opportunities.

The paper discusses also the uncertainty of lab-scale generated data for LCA. A sensitivity analysis was performed to understand how uncertainties related to lab-scale inventory data affect the life cycle impact assessment (LCIA) results and how these uncertainties would propagate during a scale-up process.

## Materials and methods

### Material and samples preparation

The feedstock represented by spruce (*Picea abies*) bark was provided as by-product from a wood processing plant. Prior to extraction, the bark was dried under normal aeration conditions. After drying, the spruce bark was milled in a GrindoMix GM 2000 equipment and the samples were vacuum packed and stored at 20 °C until extraction. The proximate analysis of biomass was performed following The Laboratory Analytical Procedures for standard biomass analysis^29^. For the whole study, the bark had 8.8% moisture and 1.8% ash content.

The total polyphenols content (TPC) was determined using the Folin-Ciocalteu method^30^. Results were expressed as milligrams gallic acid equivalents (GAE) per gram of dry spruce bark weight (TPC, mg GAE/g), calculated as follows Eq. (1):

1 $$ {\text{TPC}} = \frac{{{\text{C}}_{{{\text{GAE}}}} \cdot {\text{V}}}}{{\text{m}}} $$

where, *C*_*GAE*_ is the concentration of total polyphenols (mg GAE/mL) at a given extraction time, *V* is the volume of the extract (mL) and *m* the dry spruce bark weight (g).

### Extraction experiments

Several series of experiments for the three extraction methods were performed in order to generate comparable data. The main process parameters and polyphenols yields are presented in Table 1.

**Table 1:** Extraction parameters and polyphenols yields.

Exp no.	Extraction type	Solvent	Time (min)	Temp (°C)	TPC (mg GAE/g bark)
**Conventional extraction processes**					
1	Soxhlet ethanol extraction (SE)	Ethanol 70% v/v	394	78.37	12.39
2	Alkaline solid–liquid extraction (NaOH-SLE)	NaOH, 1%	60	90	21.66
3	UAE performed at 50 °C (UAE-50)	Ethanol 70% v/v	60	50	19.1
**UAE process alternatives**					
4	NO-UAE	Ethanol 70% v/v	60	50	11.23
5	UAE performed at 25 °C (UAE-25)	Ethanol 70% v/v	60	25	13.68
6	UAE performed with different particle sizes (UAE-PS)	Ethanol 70% v/v	60	50	2.82–29.51

Extraction with ethanol solvent (SE) was performed in a Soxhlet apparatus using 200 mL ethanol–water (70% v/v) and 10 g of spruce bark. The extraction was performed at 78.37 °C for 6 h and 34 min. After completion of extraction the solvent was recovered by distillation.

For the extraction in NaOH solution (NaOH-SLE), 3 g of spruce bark and 30 mL NaOH 1% were used. The extraction was done at 90 °C for 1 h. Afterwards, the crude ethanol extracts were separated using a Hettich Rotofix 32 centrifuge (at 4,000 rpm for 8 min) and the supernatant was carefully collected for TPC analysis.

For the UAE experiments, the extraction of polyphenols was carried out in ethanol–water (70% v/v), using a Sonorex RK 100H ultrasonic thermostatic bath (Bandelin Electronic GmbH & Co. KG, Berlin, Germany) for process intensification. The ultrasound-assisted extraction process was carried out according to the protocol previously described^31^, according to which 5 g of spruce bark were loaded in a 250 mL flat-bottom flask containing 50 mL of solvent. The flask was placed in the ultrasonic thermostatic bath operating at 35 kHz frequency and 320 W power. The temperature was maintained at 25 °C (UAE-25) and 50 °C (UAE-50), respectively (± 1 °C). The crude ethanol extracts were separated using a Hettich Rotofix 32 centrifuge (at 4,000 rpm for 4 min).

### LCA methodology

Life cycle assessment was performed considering the structure and guidelines provided by the ISO 14040 and ISO 14044 standards.

**Figure 1:**
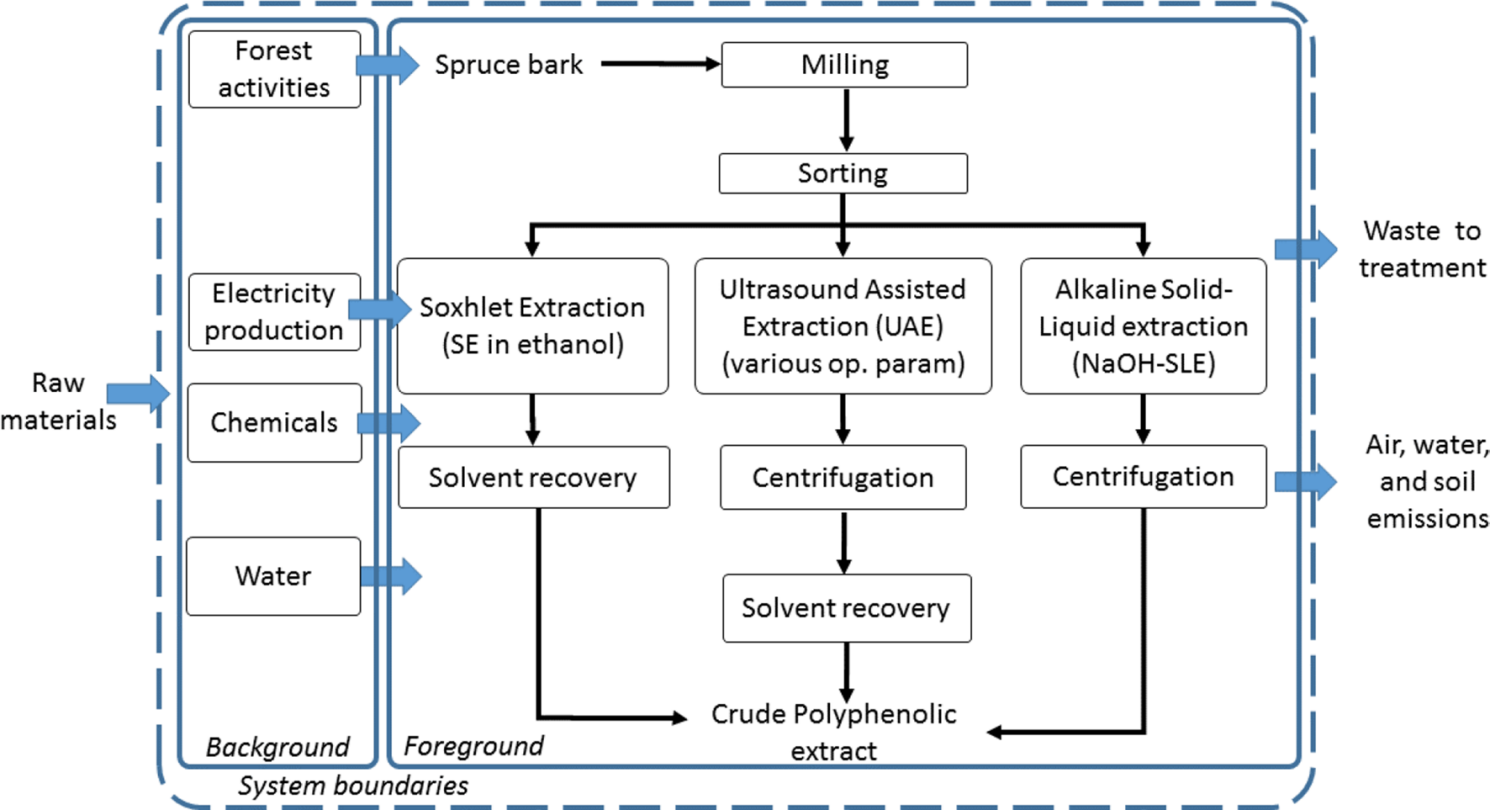
System boundaries.

The goal of this study was to compare the environmental impacts of three lab-scale extraction processes for obtaining polyphenols from spruce bark (*Picea Abies)*. The extraction methods were: a classic Soxhlet extraction using ethanol as solvent (SE), a high-temperature extraction in 1% NaOH solution (NaOH-SLE) and a non-conventional process: an ultrasound assisted extraction process (UAE) performed at 25 °C and 50 °C. Also, a UAE process applied on different particle size of bark (UAE-PS) and a blank extraction (NO-UAE) were considered. This study has a *cradle-to-gate* attributional approach considering the system boundaries presented in Fig. 1. It did not include any further polyphenols processing (like individual compound isolation), or any infrastructure processes related to the production of lab-scale equipment for the polyphenols extraction or characterization. Furthermore, our analysis did not include transport for any of the materials involved in the inventory.

The functional unit of the LCA study was defined considering the polyphenols extraction yield and it was expressed as 1 mg of polyphenols measured in mg gallic acid equivalent (mg GAE). This functional unit had enabled an effective comparison of the extraction methods, by considering also their technical efficiency expressed as polyphenols yields. All inventory entries were reported to this functional unit.

The life cycle inventory was modelled considering the quantities presented in Table 2. The input–output structure of the inventory entries, as well as their associated impacts were sourced from the Ecoinvent 3.5 database with the help of Simapro 9 software (Pre-Consultants). Electricity consumption was determined by recording the operation time of each equipment together with its power rating. For the thermo-regulated heating devices, the energy consumption was calculated for active heating (Eq. 2) and for maintaining extraction processes temperature (Eq. 3)^32^. The inventory considers the 2014 Romanian electricity mix, which features the following primary sources: 26.38% coal, 11.64% gas, 18.23% nuclear, 31.71% hydro, 9.76% wind power, 1.57% solar, 0.65% biomass. Chemical consumption inventory and solvent quantities were determined experimentally before/after each experiment.

**Table 2:** Life cycle inventory data for extraction processes (per mg polyphenols).

Inventory entry	Eco-invent process	Unit	Quantity				Data source/remarks
			Soxhlet extraction (SE)	NaOH extraction (NaOH-SLE)	UAE-50 (UAE-25)	UAE scale-up scenario	
**Inputs**							
Spruce bark	Bark chips, wet, measured as dry mass {RoW}|bark chips production, softwood, at sawmill|Alloc Rec, U	g	0.08071	0.13850	0.15707(0.07309)	0.05236	Measured
Extraction cartridge	Kraft paper, unbleached, at plant/RER U	g	0.02752				Measured
NaOH (pure)		g		0.01398			Measured extraction uses 1% NaOH solution
Ethanol (pure)	Ethanol from ethylene, at plant/RER U	g	0.89153		0.86749 (1.211)	0.00289	Measured extractions use 70% v/v ethanol in water
Electricity for heating	Electricity, low voltage {RO}| market for|Alloc Def, U	kWh	0.02750	0.061	0.0066 (0.0023)		Calculated based on power rating and operating conditions considering Eqs. 2 and 3
Heat Energy for heating	Heat, small scale , wood bark, furnace 30 kW|Alloc Def, U	MJ				0.00080	Estimated considering a bark-fired heating boiler (30 kW) with a 82% thermal efficiency
Electricity for Ultrasound generation	Electricity, low voltage {RO}|market for|Alloc Def, U	kWh			0.00314 (0.00438)	0.00010	Calculated based on power rating and operating conditions
Electricity for ethanol recovery	Electricity, low voltage {RO}|market for|Alloc Def, U	kWh	0.0011		0.0011		Calculated based on power rating and operating conditions, and Eqs. 2 and 3
Heat for Ethanol recovery energy	Heat, small scale , wood bark, furnace 30 kW|Alloc Def, U	MJ				0.00066	Estimated considering: a 95% ethanol recovery rate, a bark-fired heating boiler (30 kW) with a 82% thermal efficiency/estimated
Centrifugation energy	Electricity, low voltage {RO}|market for|Alloc Def, U	kWh		0.00148	0.00049 (0.00068)	0.00002	Calculated based on power rating and operating conditions
Water (as heat transfer medium)	Water	L	0.39548		0.15707		Measured
**Outputs**							
Polyphenols		mg	1	1	1	1	
Evaporated ethanol (air emissions)	Ethanol	g	0.17700			0.00014	Calculated for experiments from mass balance Estimated for scale-up scenario (5% losses)
Recovered ethanol	Ethanol from ethylene, at plant/RER U	g	0.71322		0.82411 (1.15062)	0.00275	Considers a 95% ethanol recovery rate
Solid waste	wood waste	g	0.08830	0.09234	0.06283 (0.08771)	0.02094	Calculated from mass balance
Waste water		L				0.00157	Estimated

2 $$Qa=m\bullet Cp\bullet \Delta T$$

3 $$Qm=hc\bullet A\bullet \Delta T$$

where, Qa is the energy required for heating (kWh), Qm is the energy for maintaining required temperature, (kWh), m is the mass of heated fluid, kg, Cp is the specific heat, (kW/kg K), hc is the global heat transfer coefficient, (W/m^2^ K), A is the heated surface area, $\Delta$ T is the temperature difference (degrees).

The life cycle impact assessment (LCIA) was performed at characterization level by using primarily ReCiPe 2016 method at midpoint level which contains 18 impact categories^33^. This method was preferred to other well established LCIA models in use (e.g. CML, Eco-Indicator, EcoScarcity) because it is one of the most updated methods from temporal and geographical standpoints, as well as from a completeness point of view. However, the LCIA results were validated with the help of other impact assessment methods like the IPCC model^34^ for climate change impacts or the UseTox 2.02^35^ for toxicity-related categories.

## Results and discussion

### Environmental profiles of extraction processes and comparison

**Figure 2:**
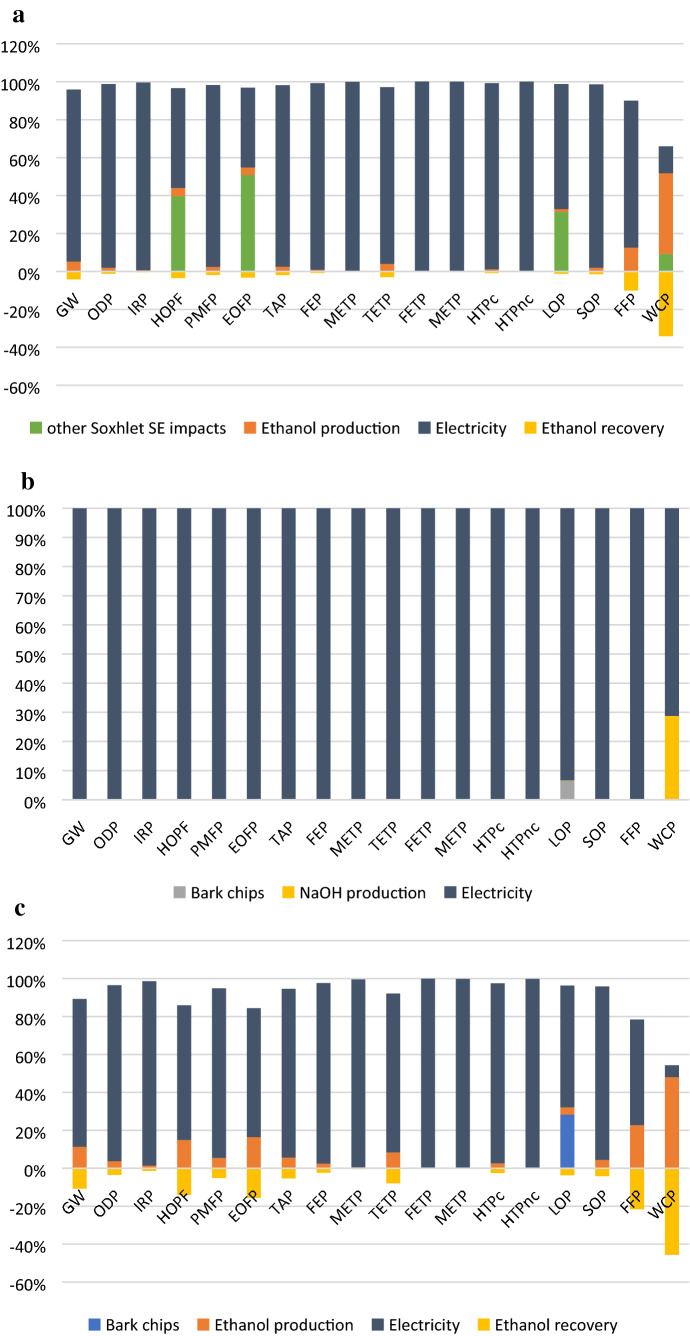
(**a**) Environmental profile of the Soxhlet extraction. (**b**) Environmental profile of NaOH LSE process. (**c**) The environmental profile of the UAE process.

The general environmental profiles for the extraction processes are presented in Table 3, where one may observe that the highest impacts in all categories are generated by the NaOH extraction, followed by the Soxhlet extraction and finally the UAE extraction. This demonstrates that for the experimental conditions used in this study, the UAE has considerably lower impacts than the other two extraction methods (Fig. 2). If we analyse the global warming impacts, for example, one may notice that the UAE extraction generates only 8 kg CO_2_ eq per gram of polyphenols, while the NaOH-SLE process generates more than five times this amount, despite the greater specific polyphenol yield of the latter process (Table 2). It is difficult to compare these results to other studies mainly due to the differences of various LCA study elements (e.g. functional units, system boundaries, product allocation and impact assessment methods) and the next citations are reported only for scaling purposes and further comparisons would not be correct. For example, considering weight as a functional unit, Ding et al.^22^ reported approximately 4 kg CO_2_ eq per kg of extracted tannins at laboratory scale, while Modahl et al.^18^ reported approximatively 1 kg CO_2_ eq per kg of extracted vanillin (at full industrial scale).

**Table 3:** Environmental profiles for the extraction processes (per functional unit of 1 mg polyphenols).

Impact category	Unit	NaOH SLE	Soxhlet extraction	UAE
Global warming	kg CO_2_ eq	43.893	20.703	8.000
Stratospheric ozone depletion	kg CFC11 eq	1.18E−05	5.57E−06	2.15E−06
Ionizing radiation	kBq Co-60 eq	14.055	6.560	2.543
Ozone formation, Human health	kg NOx eq	0.062	0.051	0.011
Fine particulate matter formation	kg PM2.5 eq	0.076	0.036	0.014
Ozone formation, Terrestrial ecosystems	kg NOx eq	0.062	0.065	0.011
Terrestrial acidification	kg SO_2_ eq	0.217	0.102	0.039
Freshwater eutrophication	kg P eq	0.085	0.040	0.015
Marine eutrophication	kg N eq	0.005	0.003	0.001
Terrestrial ecotoxicity	kg 1,4-DCB	79.615	37.494	14.471
Freshwater ecotoxicity	kg 1,4-DCB	2.448	1.141	0.443
Marine ecotoxicity	kg 1,4-DCB	3.366	1.569	0.609
Human carcinogenic toxicity	kg 1,4-DCB	4.026	1.883	0.730
Human non-carcinogenic toxicity	kg 1,4-DCB	65.293	30.440	11.821
Land use	m2a crop eq	0.378	0.259	0.092
Mineral resource scarcity	kg Cu eq	0.047	0.022	0.009
Fossil resource scarcity	kg oil eq	11.815	5.689	2.182
Water consumption	m^3^	0.776	0.585	0.138

With respect to the impacts profiles structures (Fig. 2a–c), it may be observed the most important impact contributor in all categories is electricity, followed by the solvent production. For all processes, electricity is used mainly for heating purposes (extraction processes and solvent recovery) and this generates 85.2% for UAE-50, 90% (for Soxhlet) and 95% for the NaOH-SE of the global warming impacts, for example. In case of UAE-50, the electricity consumption for ultrasound generation accounts for only 9.65% of the total impacts in the global warming category.

However, the impacts results related to energy consumption have to be interpreted with great care, because lab-scale equipment are not optimised for energy consumption and this aspect was further investigated by us in the sensitivity analysis.

Another noteworthy contributor to impact is the ethanol solvent which has a significant impact in the photochemical oxidant formation potential (HOFP and EOFP categories) due to the direct emissions during the extraction process, and the ethanol production process which impacts more significantly the Fossil resource (FFP) and water use (WCP) categories. The ethanol production-related impacts are however counteracted to some extent by recovering the ethanol (negative values in Fig. 2a,c), but this impact reduction comes with increased energy related impacts: (27.55% for UAE-50 process and 6% for the Soxhlet extraction).

The environmental profile of the extraction with NaOH solution clearly demonstrates the major contribution of the electricity consumption in the extraction process profile, as depicted in Fig. 2b.

One should consider that even if the NaOH had proven the highest polyphenol yield, its impacts are considerable higher than those of the UAE method. This proves the better environmental performance of UAE compared to the other two. It has to be pointed out that this comparison is based on data from experiments carried out in similar operational conditions.

### Influence of operational parameters

**Figure 3:**
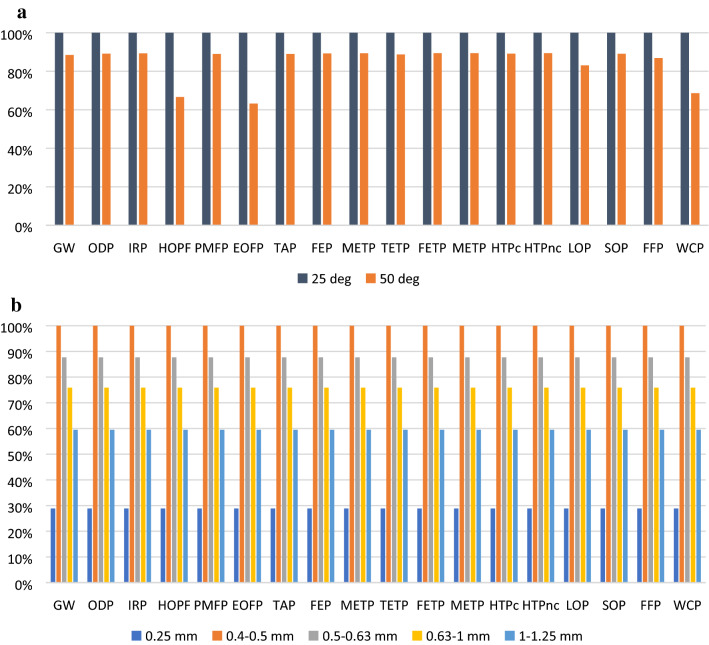
(**a**) Environmental profiles of UAE process at 25 °C and 50 °C. (**b**) Environmental profiles of UAE process for different particle sizes of spruce bark.

From Fig. 2a–c it is obvious that energy consumption is the main contributor to the total impact, in each impact category. Different operational parameters were investigated during UAE process experiments, i.e. the extraction temperature and the spruce bark particle dimensions. Comparisons of these environmental profiles are presented in Fig. 3a,b.

An increase in the extraction temperature from 25 to 50 °C leads to an increased polyphenol yield during extraction (from 16.19 mg GAE/g to 29.78 mg GAE/g) which, in turn translates to lower environmental impacts, as presented in Fig. 3a. In general, by extracting polyphenols at higher temperature leads to higher polyphenols yields, as demonstrated by Lazar et al.^31^ and this translates in a 11–12% decrease of impacts in most categories, but there are also categories where impacts decrease with 31% (water consumption), or 37% (ozone formation, terrestrial systems).

The effect of spruce bark particle size on the polyphenols yield during UAE was previously investigated by Patrautanu et al.^36^ where it was shown that the smallest particle sizes (< 0.25 mm) have had the highest polyphenol yield (29.78 mg GAE/g). For the other particle sizes in the distribution, an increase in the polyphenol yield was observed for increasing particle sizes, which is contrary to the expected effect (smaller particle size means higher surface area in contact with the extraction solvent which would lead to higher yields). This pattern is also visible in the environmental profiles presented in Fig. 3b, where the smallest impacts are due to the smallest particle sizes (< 0.25 mm), followed by particle larger than 1 mm and then impacts rise with decreasing particle size. According to Patrautanu et al.^36^ this is due to lower density of smaller particles which tend to accumulate at the surface of the ultrasonication bath and thus are not subject to the same ultrasonic energy transfer as the particles in the bulk of the liquid.

### UAE process scale-up scenario

As presented before, the main environmental impacts observed for the polyphenols extraction from spruce bark at lab scale are related to the use of electricity, mainly for heating purposes. This hotspot in the environmental profile has led to the implementation of a scenario which considers the UAE-50 process scale-up (from 3 to 300 g of input spruce bark and from 30 mL to a 30 L extraction tank) together with changing the energy source for the heating processes. For this scenario, for heating purposes, instead of using electricity (as in lab conditions), we have modelled a process that considers the use of spruce bark (kg dry mass, considering 50% moisture) as combustion fuel in a 30 kW furnace (72% efficiency). This scenario is feasible, if we consider that such a water heating boiler could exist close to a wood-processing facility. The energy output considers a lower heating value of 19 MJ/kg dry mass for the spruce bark and the emissions factors in the life cycle inventory have been updated accordingly, considering available literature^37,38^. For this scenario, other extraction parameters refer to optimal values obtained during laboratory experiments (19.1 mg GAE/g spruce bark polyphenol yield; 50 °C, 45 min extraction time, 1 mm bark particles, continuous ultrasonication regime), while other relevant inventory data related to this scenario is presented in Table 2.

**Figure 4:**
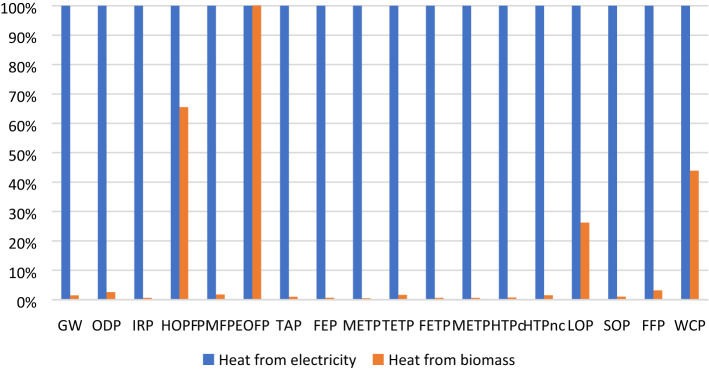
UAE-50 experimental case vs. UAE scale-up scenario comparison.

When comparing the experimental UAE-50 case (heat from electricity) with the scenario data (heat from biomass), it becomes clear that changing the heat source leads to much lower environmental impacts in most categories (this comparison is presented in Fig. 4). Of particular interest is the global warming impact which drops by 96%, mostly due to a small energy consumption, but also due to lower emissions, especially in the Global warming category (where all emissions are carbon neutral in case of biomass combustion).

**Figure 5:**
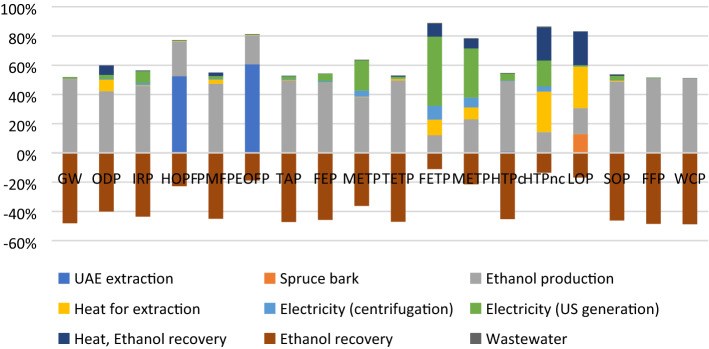
UAE process scale-up scenario profile.

The scale-up scenario generates a complex environmental impact profile (Fig. 5), which is no longer dominated by the electricity consumption for heating purposes, but rather by the ethanol used as solvent. It may be observed that by recovering the solvent, environmental benefices may appear at the expense of some heat. Furthermore, the direct ethanol emissions in the atmosphere (due to losses during extraction and recover) generate considerable impacts in ozone-depletion related categories (HODP and EODP) which suggests that it is worth implementing measures to diminish these impacts.

### Sensitivity analysis

A sensitivity analysis (SA) was performed in order to understand and evaluate the stochastic uncertainties related to how the inventory data affects the LCIA results. This is usually done by using different models for probability distributions to describe how the variability of a model input impacts the results. Although other sources of variability and hence uncertainty do exist, our analysis considers only the measured or estimated variability of key inventory flows and default variability of background processes in the inventory (default standard deviations and probability distributions of flows in the Eco-Invent data base).

The sensitivity analysis was performed by means of Monte Carlo simulations (in 10,000 points) and it was targeted to investigate the variability effect of key inventory inputs (i.e. electricity consumption, solvent use, etc.) on one of the impact categories (GW) which was considered the most important for all the extraction processes.

The SA was focused on the energy use for heating purposes, which was found to be the most important impact contributor in the GW category (Fig. 2c). Four types of variability models were investigated as SA inputs for this inventory flow: normal (two cases), uniform and triangular distributions of the probability. The lab-scale heating devices have a fairly low thermal efficiency (83%), which may be true since the lab equipment is not designed for energy efficiency, but for other purposes (parameter control, safety, etc.). Because of this, for the SA, the calculated electricity value was used as a maximum (worst-case scenario), while the minimum value of the variability interval was set to 25% of the maximum, as presented in Table 4. The mean of this range was used as mean for the centered distributions (normal 20% and triangular distributions). For the other impact contributors, normal distributions were assumed with a 10% variation around the measured value.

**Table 4:** Sensitivity analysis configuration and results.

No.	Inventory flows	Variability parameters (SA inputs)							Contribution to variance, % (SA results)			
	Input distribution type	Normal (10%)		Normal (20%)		Uniform		Triangular	Normal (10%)	Normal (20%)	Uniform	Triangular
1	Bark chips	10% around measured value							0.00%	0.10%	0.0%	0.01%
2	Ethanol production	10% around measured value							0.37%	0.34%	0.2%	1.09%
3	Electricity (for heating)	10% around calculated value		20% around mean of the interval (min–max)		max = calculated value; min = 25 of max, CV : 34.6%		max = measured value; min = 25 of max, most likely value = mean of interval (min–max),CV 24%	89.87%	88.60%	90.5%	88.59%
4	Electricity (Centrifugation)	10% around calcualated value							0.07%	0.00%	0.0%	0.33%
5	Electricity (US generation)	10% around calculated value							1.90%	0.84%	1.2%	3.27%
6	Electricity (ethanol recovery)	10% around calculated value		20% around mean of the interval (min–max)		max = calculated value; min = 25 of max, CV 31.12%		max = measured value; min = 25 of max, most likely value = mean of interval (min–max), CV 24.5%	7.46%	9.63%	7.9%	7.58%
7	Recovered ethanol	10% around measured value							0.32%	0.48%	0.3%	0.02%

The main SA results are presented in Table 4 as percent of contribution to total variability, clearly demonstrating that the main contributor to GW impacts variability and uncertainty is the electricity consumption for heating purposes. This flow contributes to the variance of these results in proportions ranging from 88.59 to 90.5%, depending on the variability model. The second contributor to the total variability of the results is the electricity for ethanol recovery.

By using these models, it was possible to predict a 95% confidence interval for each model and how much the GW impact scores would vary. The model variability, expressed by the coefficient of variation (CV), represents also a measure of the overall uncertainty for the given case. According to the CVs presented in Table 5, the SA suggests that the normal (10%) distribution generates a smaller variability (7.08%), as compared to the normal (20%) (12.48%), the triangular (15.62%) and lastly 21.78% for the uniform model. However, the SA interpretation has to consider also the probability distribution type and significance. The normal distribution describes better random phenomena for which sufficient data can be used to define a reference value (usually the mean). By contrast, the uniform distribution assesses that all values in a min–max range have the same probability of occurrence, while the triangular distribution considers besides a min–max interval, a value with the highest probability. These last two distributions types suit better our SA because they are better used in scarce-data cases, as well as in high-uncertainty situations. It has to be mentioned as well that the output coefficients of variability (7–21%) are comparable to the input ones (10–35%), or even smaller (in the case of the uniform and triangular models), which show that these models are sensible within normal limits. Thus, we may conclude that the estimated SA output values for these models (measured as CVs) are within trustful limits.

**Table 5:** Goodness of fit and statistic parameters of the SA.

Statistic parameter /case	Input distribution (for electricity for heating)			
	Normal (10%)	Normal (20%)	Uniform	Triangular
	Output distribution type (for GW, kg eq CO2)			
Distribution type (best fit)	Normal	Beta	Beta	Beta
Parameters	Mean = 19,253.74, sd = 1,363.64	Min = 6,212.11, Max = 21,169.00, Alpha = 9.11, Beta = 10.46	Min = 7,220.52, Max = 19,063.59, Alpha = 1.62,Beta = 1.52	Min = 6,168.95, Max = 18,919.97, Alpha = 4.69, Beta = 3.93
Correlation coefficient	0.9990	0.9997	0.9990	0.9959
chi square	15.812	21.612	27.992	19.06
P value chi square	0.941	0.602	0.26	0.749
most likely value	19,253.74	13,176.54	13,339.08	13,100.69
Standard Deviation (sd)	1,363.64	1,644.40	2,905.22	2,046.40
Skewness	0	0.0579	− 0.0527	− 0.1023
Kurtosis	3	2.74	2.03	2.5
Coeff. of variation (sd/most likely value*100), CV	7.08%	12.48%	21.78%	15.62%

The four probability input models have generated a good fit for all the output probability distributions as expressed by the correlation coefficients (in all cases over 0.99), as presented in Table 5. According to the chi square values and the P-value, the best fit was provided by the normal distribution (with a 10% variation coefficient), followed by the triangular, normal (20% variation coefficient) and uniform distributions. It has to be mentioned that the normal model (CV 10%) is developed around the existent electricity consumption value (mean), while the 20% CV model uses as mean the center of the min–max interval.

## Conclusions

This study had as main objective to evaluate and analyse the environmental performances of three lab-scale processes for the extraction of polyphenols from spruce bark. The analysis was carried out by using the LCA methodology which has considered the specific processes for extractions, for which relevant LCA data was measured and collected and which was related to the functional unit of the study (1 mg of extracted polyphenols measured as GAE). The LCA study has pointed out that the greatest contributor in most impact categories was the electricity used to heat the extraction systems, followed by the solvent production and emissions (for the Soxhlet and UAE processes).

An important observation is that LCA is very useful in comparing different processes by adding in to the technical and cost evaluations new and important insights related to the environmental performances. For example, the highest total polyphenols content (TPC) value was recorded for the NaOH extraction process, but this brought also the highest environmental impacts. By contrast, although the UAE process had a slightly smaller TPC yield compared to the NaOH extraction, its environmental impacts were only approximately 20% (in most impact categories) of the NaOH extraction process impacts, thus showing that environmental criteria are very important for designing and/or scaling-up extraction processes.

Another important observation is related to the fact that this LCA study was performed considering laboratory processes and equipment which are designed for research purposes when there are not always reached the optimal conditions for energy and chemicals consumption for maximizing the polyphenols extraction, as it is the case of the pilot or industrial scale systems. In this context, the scale-up scenario presented in this study is useful in demonstrating that careful scale-up design may lead to improved environmental performances.

However, further investigation is needed to include in the evaluation and analysis of the polyphenols extraction processes aspects related to the life cycle costs and environmental impacts of research, analysis and characterization equipment. A great deal of the research efforts go into these research processes and are often not considered in the technical, economic and environmental evaluation of product design or process development.

By using different input variability models it was possible to compare the different responses and uncertainties of the modeled systems. The SA has showcased that the difference in energy efficiency use between the lab-scale equipment may induce an acceptable degree of uncertainty to the GW impact results (7–21%, depending on model). Further work might be needed to expand this analysis to other impact categories and to investigate how uncertainties of LCA results obtained for lab-scale data would propagate during a scale-up process (which involves changing processes, fluxes, efficiencies and uncertainties).
